# The First Intervention Study for LGBTQ+ Older Adults With Dementia and Caregivers: COVID-19 Lessons Learned

**DOI:** 10.1093/geroni/igab046.426

**Published:** 2021-12-17

**Authors:** Karen Fredriksen Goldsen, Linda Teri, Hyun-Jun Kim, Charles Emlet, Ryan Petros, Charlotte Brown, Glenise McKenzie, David La Fazia

**Affiliations:** 1 University of Washington, Seattle, Washington, United States; 2 University of Washington, Tacoma, Washington, United States; 3 Oregon Health & Science University, Portland, Oregon, United States

## Abstract

LGBTQ+ older adults face significant health disparities with higher rates of cognitive impairment and social isolation. Yet, the cognitive health needs of LGBTQ+ adults and caregivers have not been adequately addressed in clinical trials and services. In this presentation, we will share findings from Aging with Pride: IDEA (Innovations in Dementia Empowerment and Action), the first randomly controlled trial (RCT) intervention study designed to improve quality of life of LGBTQ+ adults living with dementia and caregivers, and to reduce institutionalization. In this presentation, we will share preliminary efficacy findings, the effectiveness of culturally responsive approaches, and Covid-19 adaptations, including delivery by virtual chat rather than in-home, technology training, ensuring safety of virtual intervention components, and providing on-going technology support. Preliminary findings suggest a higher intervention retention rate in the virtual delivery as compared to in-home. This study illustrates innovative ways to serve disadvantaged communities in dementia care and aging services.

